# Cocaine

**Published:** 1890-02-22

**Authors:** 


### THERAPEUTICS.

### COCAINE.

Dr. John A. Wessinger has some observations on "The Untoward Effects of Cocaine" (*New York Medical Journal*). It should not be forgotten that cocaine is a poison, and occasionally patients present themselves, upon whom cocaine will assert its untoward effects in a most violent manner. A case is related of the injection of ten minims of a fifteen per cent. solution of cocaine hydrochloride, followed immediately by distressing dyspnœa, pallor of the face, dilated pupils, spasms of the arms and legs, small wiry pulse, and respiration increased to 40 a minute. A hypodermic injection of morphine and atropine ⅛ and 1-200 was given, and in twenty minutes the patient had sufficiently recovered for the performance of a slight operation. This experience, however, is not unique, several cases of the kind having been recorded. Dr. Wessinger asks whether cocaine manifests a poisonous action more frequently when used in certain regions of the body? We should imagine that when cocaine acts as above there must be some peculiar constitutional idiosyncracy. Many other drugs sometimes act in an unexpected manner on some constitutions, even quinine.

